# Holding the line: Provider and patient perspectives on Medications for Opioid Use Disorder continuity during the war in Ukraine

**DOI:** 10.1371/journal.pone.0341182

**Published:** 2026-02-23

**Authors:** Iryna Pykalo, Myroslava Filippovych, Oleksandr Zeziulin, Sergii Dvoriak, Lauren Dayton, Carl Latkin

**Affiliations:** 1 Ukrainian Institute on Public Health Policy, Kyiv, Ukraine; 2 Department of Health, Behavior & Society, Johns Hopkins Bloomberg School of Public Health, Baltimore, United States of America; National University, SUDAN

## Abstract

**Background:**

Ukraine has one of the highest rates of opioid use in Eastern Europe, with an estimated 346,900 people who inject drugs (PWID). Medications for opioid use disorder (MOUD) are critical for reducing opioid-related harms, including HIV transmission. However, the full-scale Russian invasion of Ukraine in February 2022 significantly disrupted MOUD services. Although the Ukraine public health system has worked to address displacement and disruptions, the ongoing armed conflict exacerbated long-standing structural challenges in the Ukrainian addiction treatment system, leading to displacement of patients and providers, damage to infrastructure, and interruptions in medication supply.

**Methods:**

This qualitative study explores the barriers and facilitators to MOUD access and retention during wartime. Semi-structured interviews were conducted between September and November 2022 with 10 MOUD patients who had been displaced due to the war and 7 healthcare providers in three Ukrainian cities (Lviv, Poltava, Ivano-Frankivsk) that received large numbers of internally displaced persons (IDPs). All patients had been enrolled in MOUD prior to the invasion and continued treatment in their new locations. Data were analyzed using the Rapid Assessment Procedure (RAP) framework to identify thematic patterns across settings and perspectives.

**Results:**

Both providers and patients reported a range of systemic, logistical, and emotional challenges affecting continuity of care. Key barriers included loss of medical records, reduced dosing due to medication shortages, delayed access for new patients, and financial hardship. Providers also described increased workloads, staff shortages, and lack of emergency preparedness. Patients often relied on peers, non-governmental organisations (NGOs), and informal channels to locate MOUD sites. Despite adversity, many patients remained committed to treatment. Providers adopted flexible, sometimes unofficial practices to sustain care delivery.

**Conclusions:**

This study highlights the fragility but also resilience of MOUD services in a conflict setting. Findings emphasize the need for policy reforms that institutionalize emergency flexibility, strengthen referral systems, support frontline staff, and integrate peer and civil society support into the national MOUD strategy. Ensuring uninterrupted access to MOUD during crises is vital for public health and for protecting the rights of people who use drugs.

## Introduction

In Ukraine, buprenorphine medication for opioid use disorder (MOUD) was first introduced in 2004 within the framework of a pilot project under the United Nations Development Programme (UNDP) to prevent HIV. In 2008, methadone was added to the MOUD programs as a treatment for opioid dependence. By the 2010s, MOUD became an integral part of the national public health strategy [1]. Alongside pharmacological treatment, comprehensive approaches integrating pharmacotherapy with psychological counseling and social interventions began to be implemented with the support of various international projects. Evaluation of these first MOUD programs showed that the program helped reduce HIV/HCV risk behaviors, decrease the use of street drugs, and improve the quality of life of patients in Ukraine [2,3].

With an estimated 346,000 people who inject drugs (PWID), 19% of whom are living with HIV [4] – Ukraine has one of the highest opioid use rates in Europe. The full-scale Russian invasion of Ukraine, that began on February 24, 2022, has significantly undermined an already strained healthcare system, particularly affecting people with chronic conditions, including MOUD treatment for people with opioid use disorder (OUD).

The number of patients receiving MOUD before the full-scale invasion was 20,331 patients (as of February 01, 2022) – 17,210 in governmental and 3,121 in private clinics. The first data available after the full-scale invasion was 16,374 patients receiving treatment in governmental MOUD clinics, as of March 01, 2022. Private clinics did not report on the number of people receiving treatment until May, 2022. As of June 01, 2022 (that is, for the full month of May 2025) 24,324 patients were receiving MOUD treatment – 18,506 in governmental clinics and 5,818 in private clinics. As of April 1, 2025, 31,974 patients were receiving MOUD services – 21,976 in governmental clinics and 9,998 in private clinics. [5]. In Ukraine, despite legislative provisions, MOUD faces challenges. Thus, accessibility remains low, covering only 5,8% of the estimated opioid-using population out of the minimum of 40% recommended by WHO, UNODC, and UNAIDS, in order to prevent and treat HIV infection among PWID [5,6]. Just 16.4% of MOUD patients have access to psychosocial support [7]. The war has intensified structural barriers to treatment, including limited medication supply, destruction of healthcare infrastructure, and the displacement of patients and providers [8].

The displacement of MOUD patients has further complicated service provision. A recent International Technology Transfer Center (ITTC) Ukraine report indicated that due to the Russian invasion, more than 7 million Ukrainians have been internally displaced within Ukraine, and 6 million left the country as of 2022, primarily from temporarily occupied territories and areas affected by active hostilities. Among them were individuals with OUD who are particularly vulnerable in resettlement, and they have faced serious challenges in maintaining treatment continuity [9]. In occupied territories, the Russian Federation has banned methadone, forcing patients into withdrawal or leading them to seek illicit alternatives [10]. This mirrors past experiences in Crimea, where MOUD services were discontinued after Russian annexation, resulting in substantial mortality and relapse rates [11]. The ongoing war in Ukraine and its escalation have exacerbated existing issues, leading to the closure of MOUD sites, disruptions in access to treatment, disruption in medication supply, and difficulties in providing psychosocial support.

One of the most pressing challenges has been the disruption of MOUD supply chains. Ukraine’s two primary methadone production facilities, located in Kharkiv and Odesa, ceased operations due to ongoing hostilities [12]. The Ukrainian Ministry of Health (MoH) responded by expanding take-home MOUD dosing from 10 to 30 days, mitigating disruptions in high-risk areas [11].

Due to destruction and disruptions near combat zones an entire set of hardships have emerged, affecting the balance between the demand and supply of MOUD and related services [7,10]: there has been an increased need for additional psychological and social support services, as well as humanitarian assistance; MOUD sites have been targeted in missile strikes by Russian Federation and closed due to destruction and occupation; problems arose with providing MOUD to conscripted patients; there is a lack of regulatory framework for the full functioning of MOUD program in the mobile treatment points format (as best practices in conflict conditions) for the distribution of narcotic drugs, including through existing mobile MOUD clinics.

Despite these challenges, there have been successful interventions aimed at sustaining MOUD services during the war. The Ukrainian Public Health Center (PHC), in collaboration with international organizations such as PEPFAR, the Alliance for Public Health, and WHO, has worked to relocate MOUD to safer regions, implement mobile outreach programs, and advocate for flexible prescribing regulations [8].

This study aims to examine the barriers and facilitators that influence MOUD enrollment and retention among patients displaced by the war. By incorporating perspectives from both patients and healthcare providers, this research seeks to identify policy strategies that can enhance MOUD accessibility in conflict settings. Given the critical role of MOUD in preventing overdose deaths and controlling the HIV epidemic, ensuring continuity of care for displaced individuals is essential for public health and human rights [10].

## Methods

This study utilizes qualitative data from semi-structured interviews to examine barriers to the uptake and adherence to MOUD following the Russian invasion of Ukraine. This research is part of a broader implementation science study focused on a peer education and social network intervention aimed at enhancing MOUD uptake and retention, as well as improving HIV medical care and antiretroviral therapy (ART) adherence among social network members living with HIV who use drugs. The semi-structured interview guides were developed by international research team (IP, MF, OZ, SD, LD, KL) in English and translated into Ukrainian and Russian for participants’ convenience.

### Participant recruitment and data collection

Participants were recruited through purposive sampling to ensure the inclusion of individuals with experience of MOUD and displacement from frontline regions. The research team engaged MOUD providers at drug treatment clinics in each selected city, providing them with inclusion criteria for eligible participants and contact details for the interviewer (IP, MF) should potential respondents wish to reach out directly. In some cases, medical staff shared participants’ contact information with interviewers after obtaining their prior consent. When interviewers directly contacted potential respondents via phone, no refusals were recorded. After patient recruitment, seven medical staff members from government clinics were also invited to participate in in-depth interviews with the research team. The distribution of provider participants by location was as follows: Lviv (three participants), Ivano-Frankivsk (one participant), and Poltava (three participants). These locations were selected because they represented regions that, at the time, had received the highest influx of internally displaced persons (IDPs), making them critical areas for assessing the accessibility and continuity of MOUD services under displacement-related pressures.

Interviews were conducted with individuals who had been receiving MOUD at governmental and private clinics before Russia’s full-scale invasion on February 24, 2022 and who continued their MOUD uptake on governmental or private sites in the cities where they relocated. The recruitment strategy aimed to ensure representation across different geographical regions, socioeconomic backgrounds and treatment experiences. The number of interviews was determined by data saturation, which was reached in each study region when no new themes emerged from subsequent interviews. Data were collected from ten patients receiving MOUD (five men and five women) at governmental clinics and who had been displaced by the war. Prior to the invasion, these participants resided in four cities in eastern Ukraine that experienced significant enemy shelling – Slovyansk, Severodonetsk, Mariupol, and Kharkiv. Following displacement, they relocated to Lviv (three participants), Ivano-Frankivsk (four participants), and Poltava (three participants). Additionally, seven MOUD providers from the same governmental clinics, where participants are receiving MOUD now, participated in the study. Data collection took place between September and November 2022.

Participants received a financial compensation of approximately 8 USD for their time.

### Ethical considerations and interview procedure

Eligibility was restricted to adults aged 18 or older who had been receiving MOUD prior to February 24, 2022. Before each interview, participants were thoroughly briefed on the study’s objectives, and oral informed consent was obtained and audio-recorded. The interviews were conducted remotely via online calls using participants’ preferred messaging applications at a convenient time. The Institutional Review Board approved the use of oral consent procedure due to the remote nature of data collection and the minimal risk involved, ensuring that participant rights and confidentiality were adequately protected.

The amount provided to participant (USD 8) was intentionally set at a modest level with the purpose of compensating participants for their time, rather than functioning as an undue incentive. To further mitigate potential bias, all participants were clearly informed that participation was entirely voluntary and that compensation would be provided regardless of the content of their responses. Confidentiality measures were strictly followed to minimize social desirability bias.

Interviews were held in Ukrainian or Russian, depending on the participant’s preference. Video conferencing was not used. Each interview, lasting approximately 40–60 minutes, was conducted by an experienced researcher with substantial expertise in qualitative interviewing of MOUD clients and providers.

The semi-structured interview guide for patients covered the following domains:

(a) Experiences with MOUD since the onset of the war
(b) Current MOUD treatment experiences
(c) Observed treatment experiences of others
(d) Other treatment experience, if any
(e) Communication with peers
(f) Barriers and facilitators of MOUD treatment

For medical providers, the interview guide included:

(a) Facility operations since the war’s onset
(b) Interaction with clients
(c) Challenges in treatment access after the full-scale invasion
(d) Intervention development questions
(e) Safety concerns for medical staff and clients

All interviews were audio-recorded and subsequently de-identified. Each respondent was assigned a study ID, which was used in all transcriptions and quotes. For accuracy and consistency, all quotes included in this study were translated into English and then back-translated into the original language by two independent research team members (IP, MF) to ensure the original meaning was preserved.

The study adhered to the qualitative research trustworthiness criterias: credibility, transferability, dependability, and confirmability [13]. We ensured credibility through triangulation and member checking; transferability through detailed contextual descriptions; dependability through maintaining a transparent audit trail; and confirmability by documenting analytic decisions and researcher reflexivity. Reporting followed COREQ/SRQR guidelines, and all ethical procedures were strictly observed [14,15].

### Data analysis

Interviews were audio-recorded and analyzed directly using descriptive summaries aligned with the domains of the data collection instrument. The analytical approach emphasized the comparison of participant experiences across different cities, culminating in structured analytical summaries [16].

For both patients and providers, data analysis followed a Rapid Assessment Procedure (RAP) framework [17], which organizes and synthesizes data based on a pre-defined analytical structure. The analytical process comprised the following steps:

(a) The data analysis team (IP, MF) reviewed two interviews to establish key terms for categorization within the RAP matrix, ensuring alignment with the study’s objectives.
(b) IP developed a preliminary categorization matrix, which was pilot-tested by the analysis team using two interview transcripts. The matrix was refined accordingly.
(c) IP and MF reviewed all interviews and summarized them using the finalized matrix, resulting in two distinct matrices encapsulating all interviews across the defined categories.
(d) The data analysis team (IP, MF, OZ) convened to identify commonalities and discrepancies within the individual matrices.
(e) IP synthesized the matrices based on team discussions, noting any disagreements. Discrepancies were reviewed iteratively by the analytical team (IP, MF, OZ) and resolved through consensus.

The final RAP matrix was utilized to compare experiences across cities and organize findings into analytical categories, which are presented in the results section alongside key healthcare system responses to the identified challenges.

## Results

### Socio-demographic characteristics

Provider participants worked in a variety of healthcare settings, including both private and governmental clinics. Four out of seven facilities specialized in addiction treatment, two (AIDS Center, Lviv and 100% Life private clinic, Poltava) out of seven specializing in HIV/AIDS and addiction treatment and care. One of the facilities (Lung Health Center (Lviv) was a multidisciplinary healthcare facility focusing on medical care for patients with tuberculosis and nonspecific respiratory diseases, as well as a full range of services for the detection and treatment of HIV, and provides MOUD for HIV-infected patients. Job responsibilities across participants are focused on healthcare provision, particularly in the field of addiction treatment and care. Six out of seven participants were MOUD providers directly involved in addiction treatment and harm reduction. One participant was a head physician, responsible for administrative and clinical oversight (Table 1).

**Table 1:** Provider socio-demographic characteristics (N = 7).

Participant	Location	Facility	Job Title
1	Poltava	100% Life private clinic	MOUD provider
2	Poltava	Governmental narcological clinic	MOUD provider
3	Lviv	Governmental narcological clinic	MOUD provider
4	Lviv	Lung Health Center	Head physician
5	Poltava	Governmental narcological clinic	MOUD provider
6	Lviv	AIDS Center	MOUD provider
7	Ivano-Frankivsk	Governmental narcological clinic	MOUD provider

The sample of MOUD patients consists of 5 males and 5 females, ensuring a balanced gender distribution among the participants. All participants were receiving methadone as part of their MOUD, except for one female participant who did not specify her drug of choice. Prior to the full-scale invasion, participants lived in different regions of Ukraine, including cities in the Donetsk oblast (such as Slovyansk, Mariupol), Luhansk oblast (Severodonetsk), and Kharkiv city. All those cities and regions were severely affected by the war with permanent heavy shelling, and one of the cities (Severodonetsk) was fully occupied by Russian forces in June 2022, leading to significant displacement among residents.

Following the invasion, participants relocated to various safer Central and Western regions of Ukraine. Four participants moved to Ivano-Frankivsk city (Western region), three to Lviv (Western region), and three to Poltava city (Central region). This forced displacement reflects the impact of the war on the participants’ ability to remain in their original locations, influencing their access to healthcare and daily lives. The duration of time that participants have been receiving MOUD varies, ranging from 2 years to 14 years, indicating a wide range of experiences with MOUD (Table 2).

**Table 2:** Patient socio-demographic characteristics (N = 10).

Participant	Drug	Location before the full-scale invasion	Location AFTER the full-scale invasion
1	methadone	Donetsk oblast, Slovyansk city	Ivano-Frankivsk
2	methadone	Kharkiv city	Lviv
3	methadone	Luhansk oblast, Severodonetsk city	Poltava
4	methadone	Kharkiv city	Poltava
5	methadone	Donetsk oblast, Mariupol city	Ivano-Frankivsk
6	methadone	Severodonetsk	Poltava
7	methadone	Donetsk oblast, Mariupol city	Lviv
8	methadone	Donetsk oblast, Mariupol city	Lviv
9	methadone	Donetsk oblast, Slovyansk city	Ivano-Frankivsk
10	methadone	Kharkiv city	Ivano-Frankivsk

## Experiences of MOUD provision and adaptation during wartime in Ukraine

The Russian-Ukrainian war has been ongoing since 2014 with a dramatic escalation following the full-scale invasion launched on February 24, 2022, which continues with sustained intensity and pressure. During this period, profound changes have occurred – not only geographically and economically, but also psychologically and socially – for all Ukrainians: those living in government-controlled areas, those residing under occupation, and those displaced to safer regions or abroad. This multifaceted burden is particularly acute for individuals who require regular or urgent medical care. Among them, patients receiving MOUD and ART are especially vulnerable, as continuous access to treatment is critical for their survival and well-being.

### 1. Structural and policy factors related to MOUD provision after the full-scale invasion

Provider participants were employed across diverse healthcare settings, covering both private and governmental clinics. Their duties were focused on addiction care and harm reduction, with one head physician overseeing administrative and clinical operations.

#### Service delivery continuity.

All service providers reported multiple challenges in ensuring uninterrupted MOUD access during the war. One of the most pressing issues was the significant increase in the number of IDPs seeking treatment. The sudden surge in demand placed additional strain on existing facilities, yet no additional medical personnel were allocated to help manage the increased workload.

*“The workload has increased significantly: several middle-level staff members left and were not replaced, while working hours remained the same despite a substantial rise in patient load.”*

MOUD provider, Poltava

#### Supply chains and local adaptations.

Another major barrier was the disruption of MOUD supply chains. Despite national stock availability, 5 out of 7 providers reported that MOUD doses were reduced by approximately 20% due to concerns about potential shortages. The MoH permitted temporarily extension of take-home doses up to 30 days; however, most clinics implemented shorter dispensing periods of 14–18 days due to logistical concerns, including safe transportation and concerns about patient adherence.

*“Although the MoH permitted take-home doses for up to 30 days, we opted to dispense only 14 days’ worth due to safety and control concerns, and also reduced the dose because of the uncertain supply situation.”*

MOUD provider, Poltava

The treatment of IDPs presented additional administrative and medical challenges. All providers reported that IDPs who arrived without medical records from their previous MOUD sites were required to be treated as new patients, meaning they had to restart treatment at lower doses, delaying stabilization.

*“IDPs without any documents are considered new patients. We start them from small doses…”*

MOUD provider, Poltava

In some regions, providers reported a complete halt in hepatitis C treatment due to a lack of rapid tests and medication supplies. Long waiting lists were another structural challenge. Patients arriving in Poltava reported delays stretching several months:

*“I came to Poltava in March, but the MOUD site put me on a waiting list — I didn’t start treatment until August.”*

Patient Kharkiv → Poltava

Furthermore, 7 out of 10 patients were unable to be officially transferred and had to undergo medical testing and re-induction from low doses.

*“If you don’t have documentation… only then they’ll let you start at the MOUD site.”*

Patient Mariupol → Lviv

Stigma and concerns about narcological registration remained significant structural barriers:

*“People are afraid to register because of police, job problems, or losing driver’s license.”*

Patient Mariupol → Lviv

#### Safety considerations.

Safety for both providers and patients remained a top priority during wartime. While no specialized protocols were issued for healthcare provision during armed conflict, providers reported receiving general recommendations from the MoH and the PHC. These included a standard algorithm of actions in the event of active hostilities in the area. One key adaptation involved increasing the number of days for take-home MOUD from a maximum of 10 days to up to 30 days in cases where the facility had to close temporarily.

*“There are no written instructions in our facility, but the administration decided that if there’s an attack on Poltava, the medical staff will give out 30 days of medication and then close the clinic.”*

*MOUD provider, Poltava*

All providers indicated that their facilities had evacuation plans; however, many faced challenges related to shelter access. Some facilities had on-site bomb shelters, while others relied on nearby shelters located within a 10-minute walking distance. Although the official guidance during air raid alerts is to leave the building and proceed to the nearest shelter, in practice, this recommendation was often disregarded. Staff typically continued dispensing medication or performing routine duties, citing reasons such as high patient volumes or the impracticality of interrupting care, especially when patients were already waiting for their MOUD doses.

### 2. Clinical and organizational factors related to MOUD provision after the full-scale invasion

#### Work load, responsibilities and location.

The highest number of IDPs receiving MOUD was reported in Lviv, the region geographically farthest from active combat zones. Although Lviv has also experienced missile strikes targeting both civilian infrastructure and critical services, the intensity of these attacks has been considerably lower compared to the eastern, northern, and southern regions of Ukraine. Providers in Lviv noted a threefold increase in the number of MOUD patients following the full-scale invasion. Despite this significant surge in patient volume, no additional support or incentives were provided to staff.

*“There were three times more people than before the war… There was no support, no additional staff, no bonuses. We worked just as we did before.”*

MOUD provider, Lviv

Providers reported no changes in the location of MOUD facilities or their core responsibilities following the full-scale invasion. However, they did experience a marked increase in patient numbers due to internal displacement, alongside a reduction in available medical personnel.

*“The workload increased a lot. Several middle-level medical staff left the clinic or even the country — around 15 people. The inpatient unit was closed, and during the war we stopped checking MOUD leftovers.”*

*MOUD provider, Poltava*

#### Stressors and coping.

Supply chain disruption and dose reductions led to withdrawal symptoms among patients, reducing treatment adherence.

Four out of seven providers reported communication challenges complicating service provision, including lack of documentation and unstable contact with displaced patients.

*“Some IDPs left the program… many started using alcohol, pills, or synthetic drugs like ‘spices’ again.”*

MOUD provider, Lviv

Providers also reported mental-health crisis among patients and lack of psychiatric support:

*“After the war started, we saw a clear increase in anxiety… we had to prescribe sleeping pills.”*

MOUD provider, Poltava

Bomb shelters were not always available, and air raid sirens interrupted care. Lack of emergency preparedness training limited organizational capacity. Despite these barriers, clinics demonstrated adaptability:

*“We extended our clinic hours — now we start as early as 7:30…”*

MOUD provider, Lviv

### 3. Interpersonal and community-level factors accessing MOUD under wartime conditions

Five out of seven providers noted peer support networks helped IDPs navigate the system:

*“Local MOUD patients really helped new IDPs — they showed them where to go…”*

MOUD provider, Poltava

Patients consistently highlighted NGO and harm reduction support as crucial:

*“At the train station, I got support from a harm reduction program.”*

Patient Slovyansk → Ivano-Frankivsk

*“A social worker met me with my medical chart and brought me to the clinic.”*

Patient Kharkiv → Ivano-Frankivsk

Harm reduction services (sterile syringes, naloxone) were available to 8 out of 10 participants and helped prevent relapse and infections.

### 4. Individual-level factors accessing MOUD under wartime conditions

#### Relocation difficulties.

Nine out of ten patients reported major psychological stress, trauma, displacement-related instability, and withdrawal during travel.

*“I didn’t have any meds, so I used pregabalin… it helped me survive.”*

Patient Mariupol → Ivano-Frankivsk

Some patients fled occupied territories, passed filtration camps, or lost homes.

*“People in Mariupol… many have died from overdoses.”*

Patient Mariupol → Lviv

Transportation difficulties were widely reported. Transportation costs, pet care, lack of income, and relocation shock limited compliance. Despite this, motivation to remain on MOUD was extremely strong.

*“I don’t need to search for drugs every day… My relationship with my family got better.”*

Patient Kharkiv → Poltava

*“I travel on foot and can only come to the clinic once every two days.”*

Patient Severodonetsk → Poltava

Participants reported varied experiences related to transferring their MOUD treatment during displacement. These ranged from smooth and rapid re-initiation of therapy in a new location to prolonged, difficult transitions marked by several days without access to MOUD. In such cases, some individuals reported using alternative substances or medications to mitigate withdrawal symptoms and cravings.

*Patient Mariupol* →*Lviv*

Another participant shared her experience of traveling without medications, including both MOUD and ART, due to fear of searches at military checkpoints. Nevertheless, thanks to support from friends, her transfer to a new city and re-initiation of MOUD was relatively smooth; she was able to begin treatment on the same day she arrived. In her new location, Poltava, she encountered financial difficulties that prevented her from affording public transportation from her place of residence to the MOUD clinic. As a result, she had to walk to the facility every other day.

*“I traveled without any medication, neither MOUD, nor ART, because I was afraid of being searched [on military checkpoints]. But the process turned out to be easy. My friends explained everything and helped with the transfer. They even brought my medical chart [to the MOUD clinic], and I was able to start treatment the same day.”*

*Patient Severodonetsk* →*Poltava*

Experiences of displacement also varied depending on the geographic origin of the participant, particularly whether their hometown was under occupation or experiencing active shelling at the time of departure. One participant, for example, described fleeing from Mariupol while it was already under Russian occupation. In addition to losing access to medical care, the participant lost their home and source of income. To reach territory controlled by Ukrainian authorities, he had to pass through a filtration camp in Mariupol—without access to medications or even basic human necessities of adequate food and shelter.

*“People in Mariupol, who don’t have the opportunity to evacuate and start MOUD, they end up using illegal drugs, and many have died from overdoses.”*

*Patient Mariupol* → *Lviv*

#### Smooth relocation.

The most seamless transfers were described by those who had prior information about which city to relocate to, where to find an MOUD site, and who carried documentation from their previous treatment facility, including the name and dosage of the prescribed medication. In contrast, participants who lacked such information or documentation faced significant delays in treatment initiation, which added distress to the already high levels of psychological stress associated with displacement and ongoing conflict.

Pre-existing patient initiative (carrying medical files, calling clinics) facilitated smooth transfers.

*“They brought my medical chart… I was able to start treatment the same day.”*

Patient Severodonetsk → Poltava

*“We were living in a school, and neighbors told us where to start MOUD. The process was easy, even though my wife didn’t have a passport. For the first two weeks, I shared my dose with her, and once she got her documents, she was able to start treatment too.”*

*Patient Kharkiv* → *Lviv*

Overall, each participant’s experience of displacement and treatment continuation was unique. Nearly all described hardship at some point—whether during the journey itself, the process of settling in a new city, or accessing essential medications. Several respondents described further complications, including a shortage of medication at MOUD sites and refusals by medical staff to initiate treatment under such conditions. Patients were sometimes placed on waiting lists for “available slots,” or experienced a deliberate reduction in their prescribed dosages. This was done by providers in an attempt to stretch limited medication supplies during uncertainty about future deliveries. As a result, some participants reported discomfort, withdrawal symptoms, and occasional use of illicit drugs to compensate for inadequate MOUD dosing.

### Discussion

This study examines the barriers and facilitators affecting MOUD access during the war in Ukraine from the perspectives of both service providers and patients. The findings highlight the significant challenges encountered in maintaining treatment services, as well as the strategies and supportive mechanisms that have helped sustain access.

The results provide an in-depth account of the lived experiences of patients and providers engaged in MOUD during the full-scale war in Ukraine. Through 17 qualitative interviews, we explored the multilevel barriers and facilitators shaping access, retention, and service adaptation across the treatment continuum. The findings confirm known structural weaknesses in the Ukrainian addiction treatment system and reveal novel dynamics introduced by wartime conditions. Below, we discuss how our results align with, expand upon, or diverge from existing literature.

### 1. Disruption and displacement as catalysts of treatment discontinuity

One of the most pronounced findings was the interruption of MOUD services due to forced displacement, regional clinic closures, and loss of continuity in care. Patients reported treatment gaps caused by evacuation without medications or documentation, mirroring earlier findings from crisis-affected regions. Our data strongly align with other research, where authors documented how Ukrainian MOUD patients displaced to European countries faced delays due to bureaucratic hurdles, geographic inaccessibility, and lack of coordination across systems [18].

Similarly, Dellamura et al. (2024) [19] emphasize that mobility and fragmentation – already common in fragile settings – are exacerbated during armed conflict, making the continuity of MOUD especially vulnerable. The challenges experienced by our participants, such as needing to re-enroll as “new patients” due to loss of medical records, are consistent with patterns observed in the HPTN 074 study, which showed that relocation often results in disengagement from HIV and MOUD services [20].

### 2. Provider overload and systemic inflexibility

Our provider interviews reflected a system pushed beyond its capacity. Staff described increased patient loads without accompanying human resource support. Clinics operated with reduced MOUD supply and hesitated to implement the MoH’s 30-day take-home policy due to fears of misuse, echoing concerns from earlier implementation studies [21,22].

This reflects what Dellamura et al. (2024) [19] call “precarious resilience” – Ukrainian MOUD programs that survived pre-war crises have some adaptive capacity but remain limited by rigid policies and chronic under-resourcing. Providers in our study employed informal coping strategies (e.g., ad hoc inter-clinic referrals or reduced dosing), reinforcing findings by Altice et al. (2022) [12] on provider ingenuity and ethical tensions under pressure.

### 3. Stigma, surveillance, and fear of narcological registration

Despite crisis conditions, structural stigma and legal anxieties continued to affect access to MOUD. Several patients voiced concern over being added to narcological registries, fearing repercussions such as loss of employment or driver’s license — an observation previously described by Makarenko et al. (2016) [22] and Carroll (2019) [23], who documented how MOUD participation in Ukraine is linked to bureaucratic surveillance.

Furthermore, patients reported social stigma from family members and peers, consistent with Bojko et al. (2015) [24], who described deeply entrenched narratives that methadone is “a life sentence”.

### 4. Critical role of peer and NGO referral pathways

In contrast to formal systems, informal and community-driven pathways emerged as essential facilitators. Nearly all patients learned about available MOUD sites through peer networks, volunteers at train stations, or NGO workers. These non-governmental actors served as intermediaries between displaced populations and overburdened clinics, echoing findings by Bojko et al. (2016) [25] and Nikitin et al. (2023) [18] on the value of peer support models and civil society in care continuity.

Patients’ reliance on unofficial channels underlines the ongoing importance of harm reduction infrastructure beyond MOUD delivery itself. In a context where formal systems are fragile or inaccessible, decentralized outreach remains a lifeline for many displaced PWID.

### 5. Innovations in crisis: Provider adaptability and micro-level solutions

Providers demonstrated notable adaptability in the face of war. While some adhered to state-issued guidelines, others deviated from protocol to maintain care – e.g., accepting verbal transfer confirmations, flexibly adjusting doses, or offering longer take-home regimens without full documentation. These micro-level innovations illustrate what Dellamura et al. (2024) [19] describe as “discretional autonomy,” where clinicians balance patient needs, structural constraints, and safety risks.

Such flexible practices reinforce the need to re-evaluate rigid policy structures. As Madden et al. (2017) [21] emphasized, successful MOUD scale-up requires systems that trust and empower providers rather than restrict them. Wartime adaptations offer a real-world test of such flexibility.

### 6. Conceptual interpretation through a socio-ecological lens

The findings of this study can be interpreted using the socio-ecological model (SEM), which conceptualizes health behaviors and treatment access as shaped by multiple, interrelated levels of influence [28]. This framework has been widely applied in addiction and HIV research to explain how individual, interpersonal, organizational, and policy-level factors collectively determine outcomes in complex systems [29].

At the individual level, MOUD patients experienced psychological distress, trauma, and financial insecurity that hindered adherence and continuity. Despite these challenges, individual motivation and perceived benefits such as reduced cravings, improved family relationships, and stable employment served as strong facilitators of retention.

At the interpersonal level, peer and family networks played a critical role in sustaining engagement. Informal support systems, including social workers and volunteers, often compensated for gaps in formal referral mechanisms. These findings underscore the importance of trust and social connectedness as protective factors during crisis.

At the organizational level, clinics demonstrated both fragility and adaptability. Providers faced increased workloads, reduced staff, and disrupted supply chains, yet many implemented flexible approaches: extended take-home doses, simplified intake, or ad hoc coordination between facilities to maintain services. These actions illustrate organizational resilience within an overstretched system.

At the policy and structural level, barriers such as documentation requirements, narcological registration, and limited national coordination revealed systemic rigidity. Conversely, temporary regulatory relaxations (e.g., extended take-home allowances) reflected an emergent adaptive capacity within Ukraine’s health system. Aligning these wartime innovations with permanent policy reform could strengthen national resilience and harmonize Ukraine’s MOUD response with global harm-reduction frameworks [6,26,27].

By situating the findings within the SEM, it becomes evident that sustaining MOUD access during conflict requires multilevel interventions addressing not only patient-level needs, but also institutional flexibility, cross-sector collaboration, and structural policy reform.

### 7. Policy implications and future directions

Findings from this study suggest several policy recommendations:

1. Codify emergency flexibility: Expand the legal basis for 30-day take-home dosing, decentralized prescribing, and mobile units—practices shown effective during wartime.
2. Protect patient confidentiality: Address registry-related fears by reforming narcological documentation and ensuring legal protections against employment or licensing discrimination.
3. Support provider well-being: Given the psychological burden and moral dilemmas faced by frontline staff, training, supervision, and safety measures must be prioritized.
4. Strengthen civil society partnerships: NGOs and peer-led initiatives have proven instrumental in reaching displaced and vulnerable populations. Their integration into the national MOUD strategy should be formalized and funded.
5. Enhance cross-regional linkage systems: To address fragmentation, there is an urgent need for a national digital registry (with secure consent protocols) allowing displaced patients to resume care without re-initiating as new patients.

The findings of this study should be interpreted within the broader context of international harm reduction and public health frameworks. The global UNAIDS 95-95-95 targets, aiming for 95% of all people living with HIV to know their status, 95% of those diagnosed to receive sustained ART, and 95% of those on ART to achieve viral suppression by 2030, cannot be achieved without adequate integration of MOUD into HIV prevention and care services. In Ukraine, only 5.8% of the estimated population of people who use opioids currently receive MOUD, falling far short of the WHO/UNODC/UNAIDS recommendation of at least 40% coverage for effective HIV prevention among people who inject drugs [5,6].

The wartime disruptions described in this study demonstrate how fragile health systems jeopardize progress toward the 95-95-95 targets [26]. Interruptions in MOUD access not only increase relapse and overdose risk but also threaten HIV care continuity, as many MOUD patients are also living with HIV and depend on integrated ART provision. Maintaining MOUD continuity under crisis conditions is thus a core component of Ukraine’s contribution to global HIV elimination goals.

Beyond HIV, the results align with the WHO and UNODC global standards for the treatment of drug use disorders, which emphasize human-rights–based and community-anchored care models [27]. The observed flexibility among Ukrainian providers, such as extended take-home dosing and peer-supported linkage mechanisms, reflects the spirit of these standards, even though many adaptations occurred informally and outside of existing regulations. Formalizing such practices in national policy could strengthen compliance with global harm-reduction principles that prioritize accessibility, dignity, and continuity of care.

### 8. Strengths and limitations

This study contributes novel insight into MOUD continuity during armed conflict through a dual-perspective approach (providers and patients). While the qualitative design allowed for rich thematic exploration, findings may not generalize to all regions or clinics in Ukraine.

Data were collected in 2022 in regions receiving internally displaced MOUD patients in areas minimally affected by the war. This sampling frame reflects the displacement patterns during the early phase of the conflict but may limit geographic representativeness. Future research should include regions not directly affected by hostilities to enhance external validity and generalizability. Additionally, the sample was limited to those who successfully re-engaged in MOUD; those who did not re-engage were not represented.

### Conclusion

The war in Ukraine has laid bare long-standing vulnerabilities in the country’s MOUD system while also showcasing resilience, adaptability, and the potential for reform. Displacement, structural stigma, and bureaucratic rigidity continue to undermine access, yet providers and patients have mobilized remarkable strategies to stay connected to care. As Ukraine’s health system evolves under pressure, these lessons offer an opportunity to reimagine MOUD not just as emergency relief but as a foundation for a more inclusive and responsive system for drug dependence care.

## Supporting information

S1 Data Patients SN MAT. (XLSX)

S2 Data Providers SN MAT. (XLSX)
